# Protocol for a scoping review study on learning plan use in undergraduate medical education

**DOI:** 10.1186/s13643-024-02553-w

**Published:** 2024-05-14

**Authors:** Anna Romanova, Claire Touchie, Sydney Ruller, Victoria Cole, Susan Humphrey-Murto

**Affiliations:** 1 The Ottawa Hospital – General Campus, 501 Smyth Rd, PO Box 209, Ottawa, ON K1H 8L6 Canada; 2 The Ottawa Hospital Research Institute, Ottawa, Canada; 3 The University of Ottawa, Ottawa, Canada; 4 The Ottawa Hospital – Riverside Campus, Ottawa, Canada

**Keywords:** Learning plan, Undergraduate medical education, Competency-based medical education, Self-regulated learning

## Abstract

**Background:**

The current paradigm of competency-based medical education and learner-centredness requires learners to take an active role in their training. However, deliberate and planned continual assessment and performance improvement is hindered by the fragmented nature of many medical training programs. Attempts to bridge this continuity gap between supervision and feedback through learner handover have been controversial. Learning plans are an alternate educational tool that helps trainees identify their learning needs and facilitate longitudinal assessment by providing supervisors with a roadmap of their goals. Informed by self-regulated learning theory, learning plans may be the answer to track trainees’ progress along their learning trajectory. The purpose of this study is to summarise the literature regarding learning plan use specifically in undergraduate medical education and explore the student’s role in all stages of learning plan development and implementation.

**Methods:**

Following Arksey and O’Malley’s framework, a scoping review will be conducted to explore the use of learning plans in undergraduate medical education. Literature searches will be conducted using multiple databases by a librarian with expertise in scoping reviews. Through an iterative process, inclusion and exclusion criteria will be developed and a data extraction form refined. Data will be analysed using quantitative and qualitative content analyses.

**Discussion:**

By summarising the literature on learning plan use in undergraduate medical education, this study aims to better understand how to support self-regulated learning in undergraduate medical education. The results from this project will inform future scholarly work in competency-based medical education at the undergraduate level and have implications for improving feedback and supporting learners at all levels of competence.

**Scoping review registration:**

Open Science Framework osf.io/wvzbx.

**Supplementary Information:**

The online version contains supplementary material available at 10.1186/s13643-024-02553-w.

## Background

Competency-based medical education (CBME) has transformed the approach to medical education to focus on demonstration of acquired competencies rather than time-based completion of rotations [1]. As a result, undergraduate and graduate medical training programs worldwide have adopted outcomes-based assessments in the form of entrustable professional activities (EPAs) comprised of competencies to be met [2]. These assessments are completed longitudinally by multiple different evaluators to generate an overall impression of a learner’s competency.

In CBME, trainees will progress along their learning trajectory at individual speeds and some may excel while others struggle to achieve the required knowledge, skills or attitudes. Therefore, deliberate and planned continual assessment and performance improvement is required. However, due to the fragmented nature of many medical training programs where learners rotate through different rotations and work with many supervisors, longitudinal observation is similarly fragmented. This makes it difficult to determine where trainees are on their learning trajectories and can affect the quality of feedback provided to them, which is a known major influencer of academic achievement [3]. As a result, struggling learners may not be identified until late in their training and the growth of high-performing learners may be stifled [4–6].

Bridging this continuity gap between supervision and feedback through some form of learner handover or forward feeding has been debated since the 1970s and continues to this day [5, 7–11]. The goal of learner handover is to improve trainee assessment and feedback by sharing their performance and learning needs between supervisors or across rotations. However, several concerns have been raised about this approach including that it could inappropriately bias subsequent assessments of the learner’s abilities [9, 11, 12]. A different approach to keeping track of trainees’ learning goals and progress along their learning trajectories is required. Learning plans (LPs) informed by self-regulated learning (SRL) theory may be the answer.

SRL has been defined as a cyclical process where learners actively control their thoughts, actions and motivation to achieve their goals [13]. Several models of SRL exist but all entail that the trainee is responsible for setting, planning, executing, monitoring and reflecting on their learning goals [13]. According to Zimmerman’s SRL model, this process occurs in three stages: forethought phase before an activity, performance phase during an activity and self-reflection phase after an activity [13]. Since each trainee leads their own learning process and has an individual trajectory towards competence, this theory relates well to the CBME paradigm which is grounded in learner-centredness [1]. However, we know that medical students and residents have difficulty identifying their own learning goals and therefore need guidance to effectively partake in SRL [14–17]. Motivation has also emerged as a key component of SRL, and numerous studies have explored factors that influence student engagement in learning [18, 19]. In addition to meeting their basic psychological needs of autonomy, relatedness and competence, perceived learning relevance through meaningful learning activities has been shown to increase trainee engagement in their learning [19].

LPs are a well-known tool across many educational fields including CBME that can provide trainees with meaningful learning activities since they help them direct their own learning goals in a guided fashion [20]. Also known as personal learning plans, learning contracts, personal action plans, personal development plans, and learning goals, LPs are documents that outline the learner’s roadmap to achieve their learning goals. They require the learner to self-identify what they need to learn and why, how they are going to do it, how they will know when they are finished, define the timeframe for goal achievement and assess the impact of their learning [20]. In so doing, LPs give more autonomy to the learner and facilitate objective and targeted feedback from supervisors. This approach has been described as “most congruent with the assumptions we make about adults as learners” [21].

LP use has been explored across various clinical settings and at all levels of medical education; however, most of the experience lies in postgraduate medical education [22]. Medical students are a unique learner population with learning needs that appear to be very well suited for using LPs for two main reasons. First, their education is often divided between classroom and clinical settings. During clinical training, students need to be more independent in setting learning goals to meet desired competencies as their education is no longer outlined for them in a detailed fashion by the medical school curriculum [23]. SRL in the workplace is also different than in the classroom due to additional complexities of clinical care that can impact students’ ability to self-regulate their learning [24]. Second, although most medical trainees have difficulty with goal setting, medical students in particular need more guidance compared to residents due to their relative lack of experience upon which they can build within the SRL framework [25]. LPs can therefore provide much-needed structure to their learning but should be guided by an experienced tutor to be effective [15, 24].

### Rationale

LPs fit well within the learner-centred educational framework of CBME by helping trainees identify their learning needs and facilitating longitudinal assessment by providing supervisors with a roadmap of their goals. In so doing, they can address current issues with learner handover and identification as well as remediation of struggling learners. Moreover, they have the potential to help trainees develop lifelong skills with respect to continuing professional development after graduation which is required by many medical licensing bodies.

An initial search of the JBI Database, Cochrane Database, MEDLINE (PubMed) and Google Scholar conducted in July–August 2022 revealed a paucity of research on LP use in undergraduate medical education (UGME). A related systematic review by van Houten–Schat et al. [24] on SRL in the clinical setting identified three interventions used by medical students and residents in SRL—coaching, LPs and supportive tools. However, only a couple of the included studies looked specifically at medical students’ use of LPs, so this remains an area in need of more exploration. A scoping review would provide an excellent starting point to map the body of literature on this topic.

The objective of this scoping review will therefore be to explore LP use in UGME. In doing so, it will address a gap in knowledge and help determine additional areas for research.

## Methods

This study will follow Arksey and O’Malley’s [26] five-step framework for scoping review methodology. It will not include the optional sixth step which entails stakeholder consultation as relevant stakeholders will be intentionally included in the research team (a member of UGME leadership, a medical student and a first-year resident).

## Step 1—Identifying the research question

The overarching purpose of this study is to “explore the use of LPs in UGME”. More specifically we seek to achieve the following:

1. Summarise the literature regarding the use of LPs in UGME (including context, students targeted, frameworks used)
2. Explore the role of the student in all stages of the LP development and implementation
3. Determine existing research gaps

## Step 2—Identifying relevant studies

An experienced health sciences librarian (VC) will conduct all searches and develop the initial search strategy. The preliminary search strategy is shown in Appendix A (see Additional file 2). Articles will be included if they meet the following criteria [27]:

- Participants
  Medical students enrolled at a medical school at the undergraduate level.
- Concept
  Any use of LPs by medical students. LPs are defined as a document, usually presented in a table format, that outlines the learner’s roadmap to achieve their learning goals [20].
- Context
  Any stage of UGME in any geographic setting.

### Types of evidence sources

We will search existing published and unpublished (grey) literature. This may include research studies, reviews, or expert opinion pieces.

### Search strategy

With the assistance of an experienced librarian (VC), a pilot search will be conducted to inform the final search strategy. A search will be conducted in the following electronic databases: MEDLINE, Embase, Education Source, APA PsycInfo and Web of Science. The search terms will be developed in consultation with the research team and librarian. The search strategy will proceed according to the JBI Manual for Evidence Synthesis three-step search strategy for reviews [27]. First, we will conduct a limited search in two appropriate online databases and analyse text words from the title, abstracts and index terms of relevant papers. Next, we will conduct a second search using all identified key words in all databases. Third, we will review reference lists of all included studies to identify further relevant studies to include in the review. We will also contact the authors of relevant papers for further information if required. This will be an iterative process as the research team becomes more familiar with the literature and will be guided by the librarian. Any modifications to the search strategy as it evolves will be described in the scoping review report. As a measure of rigour, the search strategy will be peer-reviewed by another librarian using the PRESS checklist [28]. No language or date limits will be applied.

## Step 3—Study selection

The screening process will consist of a two-step approach: screening titles/abstracts and, if they meet inclusion criteria, this will be followed by a full-text review. All screening will be done by two members of the research team and any disagreements will be resolved by an independent third member of the team. Based on preliminary inclusion criteria, the whole research team will first pilot the screening process by reviewing a random sample of 25 titles/abstracts. The search strategy, eligibility criteria and study objectives will be refined in an iterative process. We anticipate several meetings as the topic is not well described in the literature. A flowchart of the review process will be generated. Any modifications to the study selection process will be described in the scoping review report. The papers will be excluded if a full text is not available. The search results will be managed using Covidence software.

## Step 4—Charting the data

A preliminary data extraction tool is shown in Appendix B (see Additional file 3). Data will be extracted into Excel and will include demographic information and specific details about the population, concept, context, study methods and outcomes as they relate to the scoping review objectives. The whole research team will pilot the data extraction tool on ten articles selected for full-text review. Through an iterative process, the final data extraction form will be refined. Subsequently, two members of the team will independently extract data from all articles included for full-text review using this tool. Charting disagreements will be resolved by the principal and senior investigators. Google Translate will be used for any included articles that are not in the English language.

## Step 5—Collating, summarising and reporting the results 

Quantitative and qualitative analyses will be used to summarise the results. Quantitative analysis will capture descriptive statistics with details about the population, concept, context, study methods and outcomes being examined in this scoping review. Qualitative content analysis will enable interpretation of text data through the systematic classification process of coding and identifying themes and patterns [29]. Several team meetings will be held to review potential themes to ensure an accurate representation of the data. The PRISMA Extension for Scoping Reviews (PRISMA-ScR) will be used to guide the reporting of review findings [30]. Data will be presented in tables and/or diagrams as applicable. A descriptive summary will explain the presented results and how they relate to the scoping review objectives.

## Discussion

By summarising the literature on LP use in UGME, this study will contribute to a better understanding of how to support SRL amongst medical students. The results from this project will also inform future scholarly work in CBME at the undergraduate level and have implications for improving feedback as well as supporting learners at all levels of competence. In doing so, this study may have practical applications by informing learning plan incorporation into CBME-based curricula.

We do not anticipate any practical or operational issues at this time. We assembled a team with the necessary expertise and tools to complete this project.

### Supplementary Information

Additional file 1. PRISMA-P 2015 Checklist.

Additional file 2: Appendix A. Preliminary search strategy [31].

Additional file 3: Appendix B. Preliminary data extraction tool.

## Data Availability

All data generated or analysed during this study will be included in the published scoping review article.

## Abbreviations

CBME: Competency-based medical education
EPA: Entrustable professional activity
LP: Learning plan
SRL: Self-regulated learning
UGME: Undergraduate medical education
