# Supplementary material for: Protocol for a scoping review study on learning plan use in undergraduate medical education
Source: Syst Rev. 2024 May 14;13:131. doi: 10.1186/s13643-024-02553-w (PMC11095015; doi:10.1186/s13643-024-02553-w)
Supplement: Supplementary file 2 — Additional file 2: Appendix A. Preliminary search strategy [31]. [file 13643_2024_2553_MOESM2_ESM.docx]

**Appendix A. Preliminary search strategy**^*^

Ovid MEDLINE(R) ALL <1946 to November 08, 2022>

1 Students, Medical/ 41734

2 Education, Medical, Undergraduate/ 26662

3 Clinical Clerkship/ 5669

4 ((medical or medicine or clinical or doctor or doctors or physician*) adj2 (student* or trainee* or learner* or apprentic* or undergrad*)).tw,kf. 65160

5 (preclerkship* or pre-clerkship* or clerkship*).tw,kf. 6061

6 1 or 2 or 3 or 4 or 5 91089

7 self-directed learning as topic/ or programmed instructions as topic/ 2598

8 (learning adj3 (plan* or contract* or goal*)).tw,kf. 3144

9 ("personal action plan*" or "personal development plan*" or "self regulated learning" or "self directed learning").tw,kf. 2814

10 (individuali?ed adj3 (learning or plan* or contract* or goal*)).tw,kf. 4233

11 or/7-10 12331

12 6 and 11 1369

**In drafting the preliminary search strategy, the concept of “Undergraduate Medical Education” was informed by Venables’ et al^31^ scoping review protocol, a project our librarian was previously involved with.*
