# Supplementary material for: Protocol for a scoping review study on learning plan use in undergraduate medical education
Source: Syst Rev. 2024 May 14;13:131. doi: 10.1186/s13643-024-02553-w (PMC11095015; doi:10.1186/s13643-024-02553-w)
Supplement: Supplementary file 3 — Additional file 3: Appendix B. Preliminary data extraction tool. [file 13643_2024_2553_MOESM3_ESM.docx]

**Appendix B. Preliminary data extraction tool**

Article author(s):

Publication year:

Reviewer initials:

Review date:

Population

Med student level: pre-clerkship, clerkship, all years

All students vs struggling:

Concept

Formal or informal:

Who develops LP:

Student role in LP development:

Framework used for LP:

Context

Medical school:

Country:

Rotation or longitudinal:

If rotation, which one:

Why LP developed:

SRL stage LP used in:

Outcomes

Who follows up on LP:

Follow-up outcome measures:

Academic performance:

Feedback:

Student satisfaction:

Self-assessment:

Other:
